# Treatment of Depression with Botulinum Toxin

**DOI:** 10.3390/toxins14060383

**Published:** 2022-05-31

**Authors:** Marc Axel Wollmer, Michelle Magid, Tillmann H. C. Kruger, Eric Finzi

**Affiliations:** 1 Asklepios Clinic North-Ochsenzoll, Asklepios Campus Hamburg, Medical Faculty, Semmelweis University, 22419 Hamburg, Germany; 2 Dell Medical School, University of Texas at Austin, Austin, TX 78712, USA; michellemagidmd@gmail.com; 3 Department of Psychiatry, Social Psychiatry and Psychotherapy, Medical School Hannover, 30625 Hannover, Germany; krueger.tillmann@mh-hannover.de; 4 Center for Systems Neuroscience, 30559 Hannover, Germany; 5 Department of Psychiatry & Behavioral Sciences, George Washington School of Medicine, Washington, DC 20037, USA; finzieric8@gmail.com

**Keywords:** botulinum toxin, emotional proprioception, embodiment, depression, facial feedback hypothesis

## Abstract

Injection of botulinum toxin (BoNT) into the glabellar region of the face is a novel therapeutic approach in the treatment of depression. This treatment method has several advantages, including few side effects and a long-lasting, depot-like effect. Here we review the clinical and experimental evidence for the antidepressant effect of BoNT injections as well as the theoretical background and possible mechanisms of action. Moreover, we provide practical instructions for the safe and effective application of BoNT in the treatment of depression. Finally, we describe the current status of the clinical development of BoNT as an antidepressant and give an outlook on its potential future role in the management of mental disorders.

## 1. Emotional Proprioception Is a Novel Therapeutic Approach in the Treatment of Depression

Depression is one of the most common mental disorders and a leading cause of disability, affecting approximately 280 million people worldwide (WHO, https://www.who.int/news-room/fact-sheets/detail/depression accessed on 10 March 2022; Global Health Data Exchange, https://ghdx.healthdata.org/gbd-results-tool?params=gbd-api-2019-permalink/d780dffbe8a381b25e1416884959e88b accessed on 16 May 2022). Symptoms include a persistent depressed mood with feelings of sadness, irritability or emptiness as well as loss of interest and energy. Although there are effective treatments with antidepressant medications and psychotherapy, about one third of patients still suffer from chronic and/or treatment-resistant depression after several treatment trials [1]. Thus, there is a strong need for new treatment approaches. Injections of botulinum toxin (BoNT), specifically into the glabellar region, may constitute such a novel approach.

The glabellar region of the face contains the corrugator and procerus muscles. They are the mediators of frowning and thus play a key role in the facial expression of negative emotions, such as anger, fear, or sadness, which are highly prevalent in mental disorders like depression. Charles Darwin coined the term “grief muscles” for them. The combined contraction of the corrugator muscles and the medial part of the frontalis muscle produces the “omega melancholicum”, a wrinkle relief resembling the Greek letter omega (Ω), as a facial feature of emotional distress. It occurs frequently in patients suffering from mental disorders, including depression [2]. Correspondingly, a measurable over-activity of the corrugator muscles has been observed in cases of depression [3].

According to the facial feedback hypothesis, which dates back to Charles Darwin and William James in the 19th century, the facial expression of emotions generates proprioceptive feedback signals that can maintain and reinforce the expressed emotions. Through facial embodiment, an initially semi-cognitive, cool emotion may be transformed into a warmly felt emotional experience [4]. Relaxing glabellar muscles by means of BoNT injections may not only give the face a less negative/more positive expression but may also interrupt the described feedback loop and thereby confer a less negative/more positive emotional state [5]. Beyond its cosmetic benefits, the treatment, as it is applied in esthetic medicine, seems to enhance emotional wellbeing, improve social and psychological behavior, and reduce irritability, as well as depressed and anxious moods [6,7,8,9]. These effects may contribute to the popularity of treating glabellar frown lines by the injection of BoNT in esthetic medicine (https://www.isaps.org/wp-content/uploads/2022/01/ISAPS-Global-Survey_2020.pdf, accessed on 16 May 2022).

There is also experimental evidence that facial BoNT treatment can modulate the perception and processing of emotional stimuli, including the activation of the amygdala, which is a key brain structure in the processing of particularly negative emotions [9,10,11,12,13,14].

## 2. Clinical Trials Show the Efficacy of BoNT as a Treatment for Depression

Given the theoretical basis of emotional proprioception outlined above, we and others conducted several clinical studies on glabellar BoNT injections as a treatment for depression [15].

The first study was a case series of ten middle-aged women with moderate to severe depression who received a single open-label treatment with glabellar BoNT injections and showed a substantial improvement in depression scores within eight weeks, with a high response and remission rate [16].

The first randomized, double-blind, placebo-controlled trial (RCT) showed that single BoNT treatment can lead to a quick, strong and sustained improvement in depressive symptoms [17]. This trial included 30 middle-aged, mostly female patients, suffering from mild to moderate, partly chronic and treatment-resistant unipolar depression on stable treatment with antidepressant medication. The BoNT group showed a significant improvement in the symptoms of depression, while the control group remained more or less unchanged, yielding a large effect size (d = 1.28) and a response rate (>50% reduction in Hamilton Depression Rating Scale (HAM-D score)) of 60% at the primary endpoint six weeks after the baseline.

A second RCT with a larger sample (n = 74) of similar patients confirmed the antidepressant effect of BoNT [18]. In addition to similar improvement and response rates to the previous trial, the study found a significantly higher remission rate in the BoNT than in the placebo group, too.

In a third RCT with 30 patients, the initial placebo group also received BoNT after 12 weeks [19]). During the overall follow up period of 24 weeks, both groups improved significantly after BoNT treatment. Of note, the clinical improvement in depression outlasted the muscle-relaxing effect.

A fourth investigator-initiated RCT with 28 patients suffering from major depression further corroborated the antidepressant effect of BoNT treatment [20].

The Botox^®^ manufacturer Allergan conducted a multi-center phase II RCT with two doses of onabotulinumtoxinA (30 or 50 U) tested against saline placebo in 258 women diagnosed with moderate to severe depression. During the 24-week trial, only the 30 U group separated from the control group, but the numerically superior improvement narrowly missed statistical significance at the primary endpoint [21]. Basic information on the described RCTs is summarized in Table 1.

Several meta-analyses have confirmed the antidepressant effect of BoNT injected into the glabellar region. Although the number of cases examined is still quite low and the appraisal of results varies between meta-analyses, there is a high level of evidence for the efficacy of BoNT as a treatment for women with mild to moderate unipolar depression [22,23,24,25,26,27]. The safety and tolerability of the treatment was found to be excellent in all studies.

**Figure 1:**
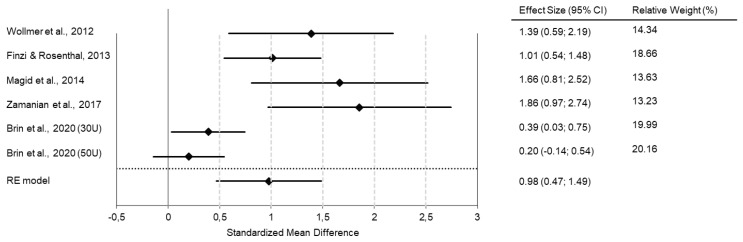
Meta-analyses of RCTs show superior antidepressant efficacy of BoNT over saline placebo injections. The forest plot shows the interaction between time (baseline vs. six weeks post-intervention) and treatment group (BoNT vs. placebo) in a combined model. All studies favor BoNT over placebo, with standard mean differences/effect sizes of Cohen’s d in a range of 0.2 to 1.86. The overall Cohen’s d of 0.98 indicates a large effect size. (Reprinted with permission from Ref. [27]). Copyright 2022 Elsevier.

In our own meta-analysis, we found an effect size of d = 0.98 when comparing treatment (BoNT vs. placebo) and time (baseline vs. 6 weeks after treatment) in one model (Figure 1). 

While RCTs have included mostly women with unipolar depression, a recent case series suggest that BoNT may be equally effective in men and in the treatment of bipolar depression [28,29].

In two comparator studies, BoNT showed at least equal efficacy and better tolerability than sertraline [30,31].

## 3. Further Studies Confirm the Antidepressant Effect of BoNT

Several conditions that are treated with BoNT, such as chronic migraine, torticollis, blepharospasm, or hyperhidrosis, are associated with high prevalence rates of comorbid depression. BoNT treatment is not only effective in the primary indications but may also alleviate the symptoms of concomitant depression [32,33,34,35,36,37,38,39,40,41,42,43,44]. Studies with BoNT in the treatment of cosmetic indications have also confirmed its antidepressant effect [45,46]. Analysis of post-marketing safety surveillance data in the FDA Adverse Event Reporting System (FAERS) revealed protective effects of BoNT against incident depression and related symptoms across various indications and injection sites [47,48].

In addition, a recent study showed antidepressant-like effects of BoNT injection in a mouse model of depression. When stress was induced by spatial restriction, mice displayed prolonged immobility times in behavioral despair tests, such as the tail suspension test and the forced swim and test, which may correspond to the experience of learned helplessness associated with depression. A single facial injection of BoNT improved this depression-like behavior and led to an increase in hippocampal serotonin levels as well as the activation of the BDNF/ERK/CREB pathway [49].

## 4. BoNT Is a Ready-to-Use Tool in the Clinical Management of Depression

Several aspects of BoNT therapy are advantageous in the management of depression. Firstly, the effect of a single treatment typically lasts three to four (two to six) months, lending BoNT the character of a “depot antidepressant”. This is practical for both patients and physicians and may enhance therapy adherence. Although BoNT is not inexpensive, BoNT therapy is cost-effective if the treatment costs are calculated per day [50]. Moreover, the safety and tolerability record of glabellar BoNT injections is excellent [51]. Transient local irritation and short episodes of headache may occur. Lid or brow ptosis is a complication that may result if glabellar BoNT injections are placed too low or too high, respectively. BoNT is not yet a registered treatment for depression and is thus off-label until it has passed phase III trials. However, patients with depression can be treated on-label for the registered indication of glabellar frown lines with the objective of attaining mood improvement as a beneficial side effect.

**Figure 2:**
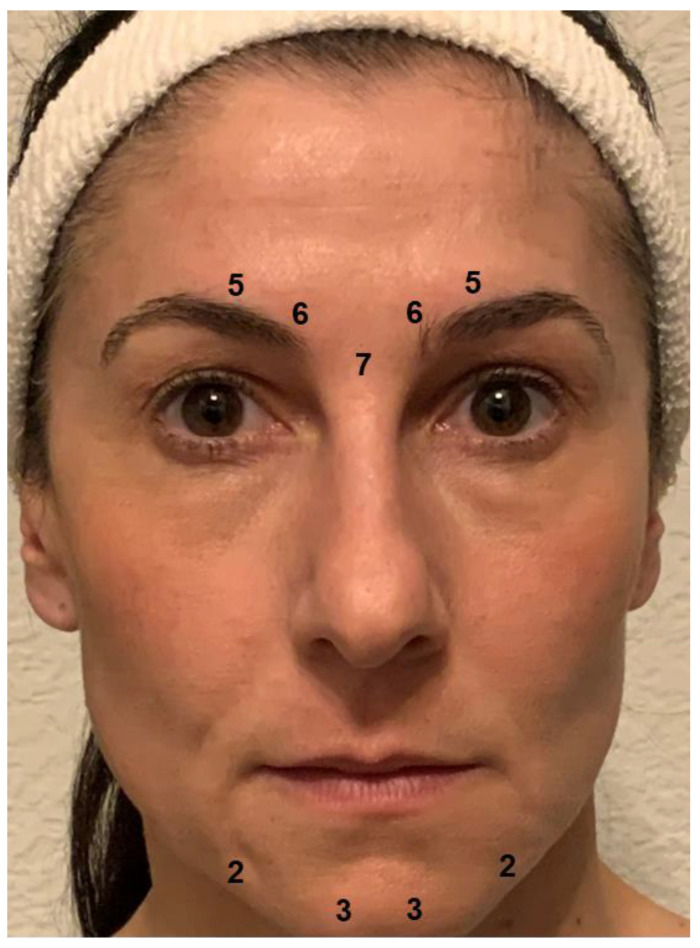
Botulinum toxin injection scheme. Most trials of BoNT for the treatment of depression used 29 units of onabotulinumtoxinA distributed to five injection points (7 U procerus muscle; 6 U corrugator muscle, medially, bilaterally; 5 U corrugator muscle, laterally, bilaterally) for women. Men received two more units at each injection point on account of their usually higher muscle mass. In clinical practice, additional injections may be placed in the chin area to preclude a mouth frown (2-3 U depressor anguli oris muscle, bilaterally; 3-5 U mentalis muscle, bilaterally).

The primary goal of BoNT injection in patients with depression is to prevent the facial expression of negative emotions and their proprioceptive reinforcement. The corrugator and procerus muscles are key in the expression of negative emotions, which generally includes a contraction of the eyebrows. Relaxing these muscles prevents the facial expression and may reduce the experience of negative emotions. BoNT-induced paralysis should be complete, as a residual activity may suffice to sustain the proprioceptive feedback loop. Therefore, the BoNT doses applied in depression may exceed those used in cosmetic treatments to obtain the desired “natural look” [52]. For women, the injection scheme used in most studies on BoNT for depression provides 29 units of onabotulinumtoxinA at a concentration of 40 or 100 U/mL 0.9% saline distributed to five injection points (7 U procerus muscle; 6 U corrugator muscle, medially, bilaterally; 5 U corrugator muscle, laterally, bilaterally; see Figure 2). Since they usually have a higher muscle mass, men received two more units at each injection point. In most cases, these doses produce complete paralysis of the glabellar musculature. In clinical practice, the doses and their distribution should be adapted to individual anatomical conditions. Particularly in patients with agitated depression, the omega melancholicum may occur. Injecting the medial part of the frontalis muscle, which is involved in its formation, is not necessary to eliminate it and can result in a cosmetically unfavorable “Mephisto” or “Spock” sign (relative lift of the lateral eyebrows).

Chin dimpling/popply chin and depression of the corners of the mouth are part of a sad facial expression and may occur habitually in depression. If this is the case, the injection of small doses of botulinum toxin into the muscles in the chin region (depressor anguli oris muscle, 2–3 U onabotulinumtoxinA, bilaterally; mentalis muscle, 3–5 U onabotulinumtoxinA, bilaterally, Figure 2) may be considered to attain a mouth-lifting effect, which may reinforce the mood-lifting effect of glabellar treatment. However, there are no data as yet from clinical studies to show that this extension of the treatment has an additional positive effect on mood. Caution is required, since overdosing or misplacement of BoNT in this region may result in the functional impairment of lip closure.

Injection of BoNT around the eyes (orbicularis oculi muscles), as applied in the treatment of crow’s feet, should be avoided in patients with depression. It may have detrimental effects on mood because these muscles are essential to the genuine Duchenne smile. Thus, their paralysis may impede both the expression and experience of happiness [53]. Accordingly, preliminary results from a trial comparing glabellar and periocular injections of BoNT as a treatment for depression indicate that glabellar injections have a superior antidepressant effect [54].

The rationale for use, the invasive dosage form, and the obviousness of the muscle-relaxing effect of BoNT injections as a treatment for depression may increase placebo effects. These are a concern in clinical studies as they hinder the delineation of the specific biological effects of BoNT from unspecific contextual influences on depressive symptomatology. However, in clinical applications, this is a clear advantage that should be used to full capacity by explaining the concept of emotional proprioception to the patients and pointing out the expected visible changes favoring a more positive facial expression to them.

BoNT treatment may be considered for “compassionate use” with depressed patients who have not sufficiently improved with or tolerated established therapies. BoNT therapy may help many patients to attain substantial improvement or even remission of depression that was previously chronic or treatment-resistant. Most patients will need regular repetition of injections to maintain an antidepressant effect, but some may stay well after a single treatment. Although the majority of the hitherto treated patients have received onabotulinumtoxinA, first impressions from treatments with equivalent doses of other BoNT species, especially incobotulinumtoxinA, are equally good.

In our clinical studies, we found numbers needed to treat between 2.2 and 4.2 for response and remission, respectively [22]. In accordance with the psychomotor concept of BoNT treatment of depression, preliminary data point to a possible positive predictive role of agitation for response [55]. An individual with major depression does not need visible frown lines to benefit from BoNT treatment [18,56]. However, there may be a weak correlation between reduction in frown lines and reduction in depression [28]. Aside from the data on agitation, there are no known predictors as to who will respond to BoNT therapy and who will not.

## 5. Several Mechanisms of Action May Account for the Antidepressant Effect of BoNT

The concept of emotional proprioception and its interruption according to the facial feedback hypothesis has been the justification for investigating BoNT as an antidepressant [15]. According to this concept, proprioceptive signals picked up by mechanical receptors in the face are conducted to the mesencephalic trigeminal nucleus and locus coeruleus. Via projections from there, they may modulate the activity of the prefrontal and insular cortex as well as of the amygdala and impact on emotional processes [15,57]. On a molecular level, facial injection of botulinum toxin can alter the metabolism of monaminergic neurotransmitters and enhance the expression of brain-derived neurotrophic factor (BDNF) in limbic brain regions [49,58].

The growing evidence for the efficacy of BoNT injection as a treatment for depression does not necessarily vindicate its rationale, nor does it show that the modulation of emotional proprioception is the one and only mechanism of action by which botulinum toxin may effect observed improvements in mood [59]. Other possible mechanisms that may be additionally or alternatively active include placebo effects as well as improved body image, self-esteem and social interactions associated with cosmetic changes [60]. In addition, animal experiments have shown axonal and even trans-synaptic transport of peripherally injected botulinum toxin into the CNS [61]. Even if botulinum toxin is not transported into the CNS in significant amounts, its peripheral action may lead to remote neuroplastic changes that may eventually account for the antidepressant effect [62]. Recent analyses of post-marketing safety surveillance data revealed protective effects of BoNT injections against incident depression across various indications and injection sites outside of the face. These observations also point to possible mechanisms of action beyond the facial feedback hypothesis and may encourage an expansion of the treated area beyond the face in future research and clinical applications.

## 6. BoNT May Play an Integrative and Transdiagnostic Role in the Treatment of Mental Disorders

The theoretical foundation of BoNT is not specific for depression but applies to any disorder that is associated with an excess in negative emotionality. Thus, the application of BoNT has a transdiagnostic potential that is already supported by auspicious case series on borderline personality disorder (BPD) and social anxiety disorder [63,64]. However, an RCT on BoNT treatment of BPD, while it showed improvement of BPD symptoms over time, did not show superiority over a control treatment with serial acupuncture [65]. Animal experiments and analyses of the FAERS database support the idea that BoNT may have a protective effect against anxiety and related disorders [66,67,68].

BoNT therapy is fundamentally different from most established psychiatric treatment approaches: conceptually, it tackles emotional processes in the CNS by their expression in the face, presumably via interruption of a reinforcing proprioceptive feedback loop. Hence, it reverses the therapeutic process from inside out (top down) to outside in (bottom up). BoNT therapy can be regarded as a pharmacologically mediated relaxation exercise that abrogates a behavior, i.e., negative facial expression, that embodies and maintains a negative emotional state. Altered facial expression may also influence emotional resonance and interaction with other people, thereby contributing to the improvement of depression. Thus, BoNT therapy integrates aspects of established treatment methods, including pharmacotherapy, relaxation exercises, behavioral therapy and social therapy.

## 7. The Clinical Development of BoNT Therapy for Depression Is Currently Stuck in Phase II

Although the numbers of patients are still low and the difficulty of reliably blinding study participants to treatment allocation represents a methodical challenge, several positive high-class RCTs and several positive meta-analyses provide good evidence for the efficacy of BoNT as a treatment for depression, specifically as an adjunctive treatment for women with mild to moderate unipolar depression. The hitherto collected data clearly justify further pursuit of this approach in phase III trials. Hence, after completion of their own phase II trials, Botox^®^ manufacturer Allergan decided in 2017 to initiate phase III of their clinical development program for BoNT as a treatment for depression. However, the trials had not started before the COVID-19 pandemic prevented the initiation of clinical trials. Subsequently, Allergan was acquired by Abbvie. Recently, an investigator-borne initiative (https://www.healisthera.com accessed on 16 May 2022) was founded to more rapidly develop BoNT therapy of depression towards registration.

## 8. Conclusions

During the last decade, a series of randomized clinical trials and meta-analyses have shown that glabellar injections of BoNT can reduce the symptoms of mild to moderate depression. Since phase III studies are still pending, there has been no registration for BoNT as a treatment for depression. However, on the basis of its registered indication in the treatment of glabellar frown lines, BoNT can be used in the clinical management of depression today, having been proven a helpful option for patients who did not improve sufficiently or who experienced side effects from treatment with established antidepressant medications. BoNT treatment targets proprioceptive feedback from the face that may have an upholding and reinforcing effect on depressed mood. However, the actual mechanisms of action are still unknown and are the subject of ongoing research.

## Figures and Tables

**Table 1:** Randomized controlled trials of BoNT as a treatment for depression.

Study	Trial Number	Participants	Intervention *	PrimaryOutcome Scale	Primary Endpoint	Main Results
[17]	NCT00934687	N = 30 (23 women/7 men)	29/39 U onabotulinumtoxinA or saline placebo	HAM-D_17_	6 weeks after baseline	Significantly greater improvement and response rate in the BoNT group
[18]	NCT01556971	N = 74 (69 women/5 men)	29/40 U onabotulinumtoxinA or saline placebo	MADRS	6 weeks after baseline	Significantly greater improvement, response and remission rate in the BoNT group
[19]	NCT01392963	N = 30 (28 women/2 men)	29/39 U onabotulinumtoxinA or saline placebo	HAM-D_21_	6 weeks after baseline	Significantly greater improvement and response rate in the BoNT group
[20]	TCTR20170409001	N = 28 (14 women/14 men)	OnabotulinumtoxinA or unspecified placebo	BDI	6 weeks after baseline	Significantly greater improvement in the BoNT group
[21]	NCT02116361	N = 123 (women only) N = 132 (women only)	30 U onabotulinumtoxinA or saline placebo 50 U onabotulinumtoxinA or saline placebo	MADRS	6 weeks after baseline	Numerically greater improvement in the 30 U BoNT group

Notes: * Doses separated by “/” refer to women and men, respectively. Abbreviations: U, units; HAM-D_17_, Hamilton Depression Rating Scale-17; MADRS, Montgomery-Asberg Depression Rating Scale; HAM-D_21_, Hamilton Depression Rating Scale-21; BDI, Beck Depression Inventory.

## Data Availability

The data presented in this study are available in this article.
